# Nomogram models for the prognosis of cervical cancer: A SEER-based study

**DOI:** 10.3389/fonc.2022.961678

**Published:** 2022-10-06

**Authors:** Kaijun Jiang, Yiqin Ai, Yanqing Li, Lianyin Jia

**Affiliations:** ^1^Yunnan Key Laboratory of Artificial Intelligence, Kunming University of Science and Technology, Kunming, China; ^2^Department of Radiation Therapy, The Third Affiliated Hospital of Kunming Medical University, Kunming, China

**Keywords:** cervical cancer, nomogram, overall survival, cancer-specific survival, SEER database

## Abstract

**Background:**

Cervical cancer (CC) is one of the most common cancers in women. This study aimed to investigate the clinical and non-clinical features that may affect the prognosis of patients with CC and to develop accurate prognostic models with respect to overall survival (OS) and cancer-specific survival (CSS).

**Methods:**

We identified 11,148 patients with CC from the SEER (Surveillance, Epidemiology, and End Results) database from 2010 to 2016. Univariate and multivariate Cox regression models were used to identify potential predictors of patients’ survival outcomes (OS and CSS). We selected meaningful independent parameters and developed nomogram models for 1-, 3-, and 5-year OS and CSS *via* R tools. Model performance was evaluated by C-index and receiver operating characteristic curve. Furthermore, calibration curves were plotted to compare the predictions of nomograms with observed outcomes, and decision curve analysis (DCA) and clinical impact curves (CICs) were used to evaluate the clinical effectiveness of the nomograms.

**Results:**

All eligible patients (n=11148) were randomized at a 7:3 ratio into training (n=7803) and validation (n=3345) groups. Ten variables were identified as common independent predictors of OS and CSS: insurance status, grade, histology, chemotherapy, metastasis number, tumor size, regional nodes examined, International Federation of Obstetrics and Gynecology stage, lymph vascular space invasion (LVSI), and radiation. The C-index values for OS (0.831 and 0.824) and CSS (0.844 and 0.841) in the training cohorts and validation cohorts, respectively, indicated excellent discrimination performance of the nomograms. The internal and external calibration plots indicated excellent agreement between nomogram prediction and actual survival, and the DCA and CICs reflected favorable potential clinical effects.

**Conclusions:**

We constructed nomograms that could predict 1-, 3-, and 5-year OS and CSS in patients with CC. These tools showed near-perfect accuracy and clinical utility; thus, they could lead to better patient counseling and personalized and tailored treatment to improve clinical prognosis.

## Introduction

Cervical cancer (CC) is one of the most commonly occurring cancers in women. Despite being one of the most preventable cancers through screening, cervical cancer caused the death of 4138 women in the US in 2018, the equivalent of 11 women per day, one-half of whom were aged ≤58 years at death (1). Although the overall incidence of CC has been declining for decades, rates of the distant-stage disease and cervical adenocarcinoma, which are often undetected by cytology, are increasing; this increase is largely driven by trends in young women (2). These findings underscore the need for more targeted efforts to increase both human papillomavirus (HPV) vaccination among all individuals aged ≤26 years and primary HPV testing or HPV/cytology co-testing every 5 years in women from the age of 25 years, as recommended by the American Cancer Society in updated guidelines published in 2020 (3, 4). The clinical stage is a reliable and widely accepted indicator that can be used to evaluate the prognosis of patients with CC (5). At the end of April 2022, there were two main clinical staging schemes: the American Joint Committee on Cancer (AJCC) Staging Seventh Edition and the International Federation of Obstetrics and Gynecology (FIGO) 2009 Guidelines. FIGO staging is mainly based on clinical characteristics, but few studies have considered the impact of non-clinical parameters on its clinical utility and net benefit in patients with CC (6–9). Clinical staging is mainly based on cervical tumor size or extent of pelvic disease, with less weight given to other important prognostic factors such as age, race, and treatment modality. Therefore, clinical staging alone is insufficient to predict the prognosis of patients with CC, and a more complete prognostic assessment protocol is required. Herein, we revised the TNM stage according to the FIGO classification (2009 version) and explored the use of nomogram models for prognosis prediction in patients with CC in terms of overall survival (OS) and cancer-specific survival (CSS) in combination with clinical and non-clinical indicators.

The nomogram model is a simple visualization tool based on multivariate Cox proportional hazard regression, which is becoming increasingly popular in oncology as a means of predicting and quantifying the probability of an individual patient’s survival (10). Our data were based on the Surveillance, Epidemiology, and Outcomes Database (SEER), which collects data on cancer diagnosis, treatment, and survival, covering approximately 35% of the US population (11). This widely used resource collects demographic, clinical, and outcome information on all types of cancer and makes it freely available to researchers (11).

In this retrospective study, we developed nomogram models to provide a simple graphical representation of clinical events and generate numerical probabilities (12), and derived and validated prognostic profiles to predict OS and CSS in patients with CC enrolled in the SEER database from 2010 to 2016. We expect these nomograms to have applications in supporting clinical decision-making and ongoing work. Compared with other studies evaluating survival prognosis in patients with CC using nomograms (6–9), the sample of patients with more complete patient parameters enrolled in our study enabled us to use more real-time data (13). Importantly, we predicted CSS and OS and evaluated our model internally and externally using five approaches: the C-index (Harrell protocol index), receiver operating characteristic (ROC) curves, calibration plots, decision curve analysis (DCA) and clinical impact curves (CICs), making our study more complete and reliable compared with previous studies.

## Material and methods

### Patients and endpoints

The study used the database of the SEER National Cancer Institute (https://seer.cancer.gov/), a free US cancer registry. We gained access to SEER database files, and all authors followed SEER database policies throughout the search process. Individual informed consent was not required because no personal data were used in this study.

Information on patients newly diagnosed with CC between 2010 and 2016 was extracted from the SEER-18 database using the SEER^∗^Stat software version 8.3.9.2. As information on site-specific metastases was only available from 2010 in the SEER database, we limited the scope of the analysis to the period 2010–2016. Patients with CC were considered eligible to be enrolled in this study if they had only one primary malignancy, an end date of active surveillance, and complete clinical and pathological information (e.g., age, race, FIGO stage, tumor grade, and treatment). Variables for each patient included age, race, marital status, insurance, primary site, TNM status, pathological type, histological grade, distant metastasis, treatment strategy, vital status, and survival time. The exclusion criteria in this study were as follows: (a) unknown AJCC 6th TNM stage; (b) unknown marital status; (c) unknown race; (d) regional median family income; (e) unknown laterality of the tumor; (f) unknown tumor size; (g) unknown histological grade; (h) unknown radiotherapy and chemotherapy records; and (i) unknown survival months. In our study, TNM status was classified according to FIGO (2009 edition). Distant metastases were diagnosed in the lymph nodes, liver, lung, bone, and brain. We added a variable of “metastasis numbers”, which was classified according to the transfer of organs. Local treatment of primary tumors was mainly by surgery or radiation therapy. The surgical approach was characterized by three variables: radiation sequence with surgery (RSS), primary site surgery, and regional lymph node surgery (RLNS). Radiation was classified into four types: beam radiation, brachytherapy, combination of beam with brachytherapy (BRB), and no/unknown treatment. The primary endpoints of this study were OS and CSS of patients with CC.

### Statistical analysis

All eligible patients were randomized in a 7:3 ratio into training and validation groups. Chi-square test was used to compare clinical and pathological characteristics between the training and validation groups. The nomograms were developed in the training cohort as follows. First, univariate Cox analysis was used to evaluate the ability of each variable to predict OS. Second, variables that reached statistical significance in the univariate Cox analysis were fitted in the multivariate Cox analysis. To identify independent variables that had significant impact on patient outcomes, an adverse selection procedure using Akaike information criterion (AIC) scores for variable selection was introduced. Finally, the remaining variables were used in the construction of the nomograms. The primary endpoints of the nomograms were 1-, 3-, and 5-year OS and CSS.

The nomograms were validated in the training cohort and the validation cohort. To assess the predictive accuracy of the nomogram, we used the C-index, ROC curves (14), and calibration curves (with 1000 bootstrap resamples) to visually differentiate the predicted and actual values for 1-, 3-, and 5-year OS and CSS. Furthermore, DCA and CICs were used to assess the clinical value of the nomogram (15). Kaplan–Meier analysis and log-rank test were used to investigate the differences in survival between three risk subgroups. The chi-square test results for these variables between the training and validation cohorts all had P > 0.05. All analyses were conducted using R version 4.1.3 in RStudio.

## Results

### Patient baseline characteristics

Our study identified 11,148 eligible patients diagnosed with CC from 2010 to 2016, with 7803 patients assigned to the training cohort and 3345 patients to the validation cohort. The demographic and clinicopathologic characteristics of patients in the training and validation cohorts are listed in **Table 1**. The median age of all patients was 47 years, with a range of 20–70 years. Most patients in both cohorts were older (≥40 years) and White. The most common pathological type of CC was SCC (squamous cell carcinoma) (69.62%). Regarding metastasis (5.10%), the most frequent site of metastasis was the lung (3.23%), followed by bone (1.96%), liver (1.52%), and brain (0.31%). In both cohorts, more than half of the patients were treated with radiotherapy or chemotherapy. In addition, initial examination of regional lymph nodes had been performed for only about 45% of patients.

**Table 1:** Characteristics of patients with cervical cancer in the training cohort and validation cohort.

Characteristic	Training cohort	Validation cohort	All subjects	*P*
	7803 (70)	3345 (30)	11148 (100)	
Age, median [range]	47 [38, 56]	47 [38, 57]	47 [38, 56]	0.9977
Age, *n* (%)				0.7714
>=20 and <40	2198 (28.17)	952 (28.46)	3150 (28.26)	
>=40 and <70	5605 (71.83)	2393 (71.54)	7998 (71.74)	
Race, *n* (%)				0.2534
Black	1025 (13.14)	416 (12.44)	1441 (12.93)	
White	5953 (76.29)	2600 (77.73)	8553 (76.72)	
Other	825 (10.57)	329 (9.84)	1154 (10.35)	
Marital, *n* (%)				0.6559
Married	5177 (66.35)	2204 (65.89)	7381 (66.21)	
Single	2626 (33.65)	1141 (34.11)	3767 (33.79)	
Insurance, *n* (%)				0.2202
Insured	7301 (93.57)	3108 (92.91)	10409 (93.37)	
Uninsured	502 (6.43)	237 (7.09)	739 (6.63)	
Primary site, *n* (%)				0.5019
Cervix uteri	5971 (76.52)	2520 (75.34)	8491 (76.17)	
Endocervix	1556 (19.94)	708 (21.17)	2264 (20.31)	
Exocervix	141 (1.81)	57 (1.70)	198 (1.78)	
OLC	135 (1.73)	60 (1.79)	195 (1.75)	
Grade, *n* (%)				0.9313
Grade I	1238 (15.87)	531 (15.87)	1769 (15.87)	
Grade II	3385 (43.38)	1466 (43.83)	4851 (43.51)	
Grade III	2968 (38.04)	1253 (37.46)	4221 (37.86)	
Grade IV	212 (2.72)	95 (2.84)	307 (2.75)	
Histology, *n* (%)				0.7464
SCC	5443 (69.76)	2318 (69.30)	7761 (69.62)	
AC	1952 (25.02)	858 (25.65)	2810 (25.21)	
Other	408 (5.23)	169 (5.05)	577 (5.18)	
RSS, *n* (%)				0.4703
No	5569 (71.37)	2364 (70.67)	7933 (71.16)	
Yes	2234 (28.63)	981 (29.33)	3215 (28.84)	
Chemotherapy, *n* (%)				0.8528
Yes	4110 (52.67)	1769 (52.88)	5879 (52.74)	
No	3693 (47.33)	1576 (47.12)	5269 (47.26)	
Bone metastasis, *n* (%)				0.0979
No	7662 (98.19)	3268 (97.70)	10930 (98.04)	
Yes	141 (1.81)	77 (2.30)	218 (1.96)	
Brain metastasis, *n* (%)				0.7113
No	7777 (99.67)	3336 (99.73)	11113 (99.69)	
Yes	26 (0.33)	9 (0.27)	35 (0.31)	
Liver metastasis, *n* (%)				0.4488
No	7689 (98.54)	3289 (98.33)	10978 (98.48)	
Yes	114 (1.46)	56 (1.67)	170 (1.52)	
Lung metastasis, *n* (%)				0.3818
No	7559 (96.87)	3229 (96.53)	10788 (96.77)	
Yes	244 (3.13)	116 (3.47)	360 (3.23)	
Metastasis numbers, *n* (%)				0.3610
0	7415 (95.03)	3164 (94.59)	10579 (94.90)	
1	271 (3.47)	120 (3.59)	391 (3.51)	
2	97 (1.24)	46 (1.38)	143 (1.28)	
>=3	20 (0.26)	15 (0.45)	35 (0.31)	
Tumor size, *n* (%)				0.4562
<4 cm	7158 (64.21)	2130 (63.68)	5028 (64.44)	
>=4 cm	3990 (35.79)	1215 (36.32)	2775 (35.56)	
Regional nodes examined, *n* (%)				0.0484
No	4160 (53.31)	1839 (54.98)	5999 (53.81)	
Yes	3614 (46.32)	1501 (44.87)	5115 (45.88)	
UNK	29 (0.37)	5 (0.15)	34 (0.30)	
Regional nodes positive, *n* (%)				0.0588
No	2773 (35.54)	1176 (35.16)	3949 (35.42)	
negative	4160 (53.31)	1839 (54.98)	5999 (53.81)	
positive	838 (10.74)	324 (9.69)	1162 (10.42)	
UNK	32 (0.41)	6 (0.18)	38 (0.34)	
FIGO, *n* (%)				0.4472
IA1	875 (11.21)	397 (11.87)	1272 (11.41)	
IA2	333 (4.27)	137 (4.10)	470 (4.22)	
IB1	2196 (28.14)	939 (28.07)	3135 (28.12)	
IB2	706 (9.05)	334 (9.99)	1040 (9.33)	
IIA	529 (6.78)	244 (7.29)	773 (6.93)	
IIB	1206 (15.46)	479 (14.32)	1685 (15.11)	
IIIA	229 (2.93)	99 (2.96)	328 (2.94)	
IIIB	865 (11.09)	356 (10.64)	1221 (10.95)	
IVA	296 (3.79)	135 (4.04)	431 (3.87)	
INOS	461 (5.91)	179 (5.35)	640 (5.74)	
IINOS	14 (0.18)	11 (0.33)	25 (0.22)	
IIINOS	93 (1.19)	35 (1.05)	128 (1.15)	
LVSI, *n* (%)				0.0931
No	5634 (72.20)	2457 (73.45)	8091 (72.58)	
Yes	2001 (25.64)	835 (24.96)	2836 (25.44)	
UNK	168 (2.15)	53 (1.58)	221 (1.98)	
Primary site surgery, *n* (%)				0.1676
No	4189 (53.68)	1844 (55.13)	6033 (54.12)	
Yes	3614 (46.32)	1501 (44.87)	5115 (45.88)	
RLNS, *n* (%)				0.2077
No	4191 (53.71)	1853 (55.40)	6044 (54.22)	
Yes	3549 (45.48)	1470 (43.95)	5019 (45.02)	
UNK	63 (0.81)	22 (0.66)	85 (0.76)	
Radiation, *n* (%)				0.9049
Beam radiation	2273 (29.13)	969 (28.97)	3242 (29.08)	
brachytherapy	10 (0.13)	4 (0.12)	14 (0.13)	
BRB	2235 (28.64)	981 (29.33)	3216 (28.85)	
No/UNK	3285 (42.10)	1391 (41.58)	4676 (41.94)	
months, median [range]	30 [16, 53]	30 [16, 53]	30 [16, 53]	0.8927

FIGO, International Federation of Gynecologists and Obstetricians; UNK, Unknown; OLC, overlap crossing; RSS, Radiation sequence with surgery; AC, adenocarcinoma; SCC, Squamous cell carcinoma; FIGO, the International Federation of Gynecology and Obstetrics; LVSI, lymph vascular space invasion; RLNS, Regional lymph node surgery; BRB, Combination of beam with brachytherapy.

### Univariate and multivariate analyses

The results of the univariate and multivariate Cox regression analyses (16) for OS and CSS in the training cohort are shown in **Table 2**. In the univariate Cox regression, all variables were significant for both OS and CSS (P < 0.05). Therefore, all variables were included in the multivariate Cox regression analyses for OS and CSS to identify independent prognostic factors. For OS, the independent prognostic factors included age, race, insurance, grade, histology, chemotherapy, metastasis number, tumor size, regional nodes examined, FIGO stage, lymph vascular space invasion (LVSI), regional lymph node surgery (RLNS), and radiation. For CSS, the independent prognostic factors included marital status, insurance, grade, histology, chemotherapy, metastasis number, tumor size, regional nodes examined, FIGO stage, LVSI, and radiation. Compared with the independent for OS, these findings of CSS were not consistent in terms of independent prognostic variables including age, race, marital status, and RLNS.

**Table 2:** Univariate and multivariate Cox regression analysis of OS and CSS in cervical cancer (training cohort).

			OS				CSS			
Variables	Reference	Characteristic	Univariate Cox		Multivariate Cox		Univariate Cox		Multivariate Cox	
			HR	*P*	HR	*P*	HR	*P*	HR	*P*
Age	>=20 and <40	>=40 and <70	1.93 (1.72 - 2.16)	**<0.001**	1.21 (1.08 - 1.36)	**0.0015**	1.66 (1.47 - 1.87)	**<0.001**	1.02 (0.9 - 1.16)	0.7322
Race	Black	White	0.63 (0.56 - 0.7)	**<0.001**	0.82 (0.73 - 0.93)	**0.0013**	0.67 (0.59 - 0.76)	**<0.001**	0.9 (0.79 - 1.03)	0.1201
	Black	Other	0.62 (0.52 - 0.74)	**<0.001**	0.81 (0.68 - 0.97)	**0.0199**	0.7 (0.57 - 0.84)	**<0.001**	0.93 (0.77 - 1.14)	0.4912
Marital	Married	Single	1.2 (1.09 - 1.31)	**<0.001**	1.06 (0.96 - 1.16)	0.2557	1.28 (1.16 - 1.41)	**<0.001**	1.12 (1.01 - 1.25)	**0.031**
Insurance	Insured	Uninsured	1.59 (1.36 - 1.86)	**<0.001**	1.24 (1.06 - 1.45)	**0.0086**	1.7 (1.44 - 2.01)	**<0.001**	1.35 (1.13 - 1.6)	**<0.001**
Primary site	Cervix uteri	Endocervix	0.6 (0.53 - 0.68)	**<0.001**	0.99 (0.86 - 1.14)	0.8986	0.6 (0.52 - 0.69)	**<0.001**	1.02 (0.87 - 1.19)	0.8149
	Cervix uteri	Exocervix	0.62 (0.41 - 0.93)	**0.02**	0.83 (0.55 - 1.24)	0.3641	0.71 (0.47 - 1.08)	0.11	0.99 (0.65 - 1.51)	0.9557
	Cervix uteri	OLC	0.97 (0.7 - 1.34)	0.835	1.23 (0.89 - 1.72)	0.2111	1.08 (0.76 - 1.53)	0.661	1.37 (0.97 - 1.95)	0.0776
Grade	Grade I	Grade II	2.61 (2.14 - 3.18)	**<0.001**	1.32 (1.07 - 1.62)	**0.0091**	2.88 (2.28 - 3.64)	**<0.001**	1.35 (1.06 - 1.72)	**0.017**
	Grade I	Grade III	4.66 (3.84 - 5.66)	**<0.001**	1.72 (1.4 - 2.11)	**<0.001**	5.49 (4.36 - 6.92)	**<0.001**	1.84 (1.45 - 2.35)	**<0.001**
	Grade I	Grade IV	6.82 (5.2 - 8.94)	**<0.001**	2.48 (1.86 - 3.3)	**<0.001**	7.97 (5.85 - 10.87)	**<0.001**	2.64 (1.91 - 3.66)	**<0.001**
Histology	AC	SCC	1.77 (1.58 - 2)	**<0.001**	1.09 (0.95 - 1.25)	0.222	1.78 (1.56 - 2.04)	**<0.001**	1.09 (0.93 - 1.27)	0.2706
	AC	Other	3.33 (2.78 - 4)	**<0.001**	1.55 (1.28 - 1.89)	**<0.001**	3.57 (2.93 - 4.37)	**<0.001**	1.6 (1.3 - 1.99)	**<0.001**
RSS	No	Yes	0.72 (0.65 - 0.8)	**<0.001**	1.05 (0.92 - 1.2)	0.4688	0.74 (0.67 - 0.83)	**<0.001**	1.04 (0.9 - 1.21)	0.582
Chemotherapy	No	Yes	2.81 (2.54 - 3.1)	**<0.001**	0.61 (0.54 - 0.69)	**<0.001**	3.06 (2.73 - 3.43)	**<0.001**	0.59 (0.52 - 0.68)	**<0.001**
bone metastasis	No	Yes	11.46 (9.54 - 13.76)	**<0.001**	0.89 (0.52 - 1.5)	0.6565	12.03 (9.88 - 14.64)	**<0.001**	0.71 (0.4 - 1.27)	0.2497
brain metastasis	No	Yes	17.97 (12.16 - 26.6)	**<0.001**	1.46 (0.76 - 2.79)	0.2555	17.84 (11.67 - 27.27)	**<0.001**	1.13 (0.56 - 2.31)	0.7291
liver metastasis	No	Yes	11.02 (9.01 - 13.47)	**<0.001**	0.85 (0.5 - 1.44)	0.5441	12.31 (9.98 - 15.19)	**<0.001**	0.74 (0.41 - 1.31)	0.3012
lung metastasis	No	Yes	9.01 (7.8 - 10.42)	**<0.001**	0.69 (0.4 - 1.18)	0.1708	9.72 (8.32 - 11.35)	**<0.001**	0.57 (0.31 - 1.03)	0.0626
Metastasis numbers	>=3	0	0.05 (0.03 - 0.08)	**<0.001**	0.12 (0.04 - 0.37)	**<0.001**	0.06 (0.03 - 0.09)	**<0.001**	0.08 (0.02 - 0.26)	**<0.001**
	>=3	1	0.46 (0.29 - 0.73)	**0.001**	0.43 (0.23 - 0.81)	**0.0094**	0.51 (0.3 - 0.86)	**0.012**	0.34 (0.17 - 0.67)	**0.002**
	>=3	2	0.91 (0.55 - 1.49)	0.698	NA	NA	1.09 (0.63 - 1.89)	0.758	NA	NA
Tumor size	<4 cm	>=4 cm	2.68 (2.45 - 2.93)	**<0.001**	1.14 (1.03 - 1.26)	**0.0121**	2.92 (2.65 - 3.22)	**<0.001**	1.14 (1.02 - 1.28)	**0.02**
Regional nodes examined	No	Yes	0.29 (0.26 - 0.32)	**<0.001**	0.57 (0.41 - 0.79)	**<0.001**	0.29 (0.26 - 0.33)	**<0.001**	0.55 (0.38 - 0.79)	**0.001**
	No	UNK	1.85 (1.15 - 2.98)	**0.012**	50639.75 (0 - Inf)	0.9816	2.33 (1.44 - 3.75)	**0.001**	44977 (0 - Inf)	0.9841
Regional nodes positive	negative	No	5.83 (5.07 - 6.71)	**<0.001**	NA	NA	6.15 (5.24 - 7.23)	**<0.001**	NA	NA
	negative	positive	4.16 (3.47 - 4.98)	**<0.001**	1.96 (1.57 - 2.44)	**<0.001**	4.71 (3.84 - 5.76)	**<0.001**	1.83 (1.43 - 2.33)	**<0.001**
	negative	UNK	8.87 (5.42 - 14.52)	**<0.001**	0 (0 - Inf)	0.9817	11.84 (7.19 - 19.5)	**<0.001**	0 (0 - Inf)	0.9844
FIGO	IA1	IA2	1.85 (0.93 - 3.69)	0.08	2.54 (1.27 - 5.08)	**0.0085**	1.76 (0.67 - 4.63)	0.25	2.23 (0.85 - 5.89)	0.1041
	IA1	IB1	3.62 (2.25 - 5.81)	**<0.001**	5.51 (3.39 - 8.97)	**<0.001**	4.37 (2.29 - 8.35)	**<0.001**	6 (3.1 - 11.61)	**<0.001**
	IA1	IB2	13.73 (8.57 - 22.01)	**<0.001**	15.13 (9.2 - 24.88)	**<0.001**	21.95 (11.59 - 41.59)	**<0.001**	22.03 (11.35 - 42.8)	**<0.001**
	IA1	IIA	19.38 (12.08 - 31.1)	**<0.001**	18.5 (11.3 - 30.28)	**<0.001**	28.5 (15.01 - 54.1)	**<0.001**	25.22 (13.03 - 48.8)	**<0.001**
	IA1	IIB	17.98 (11.34 - 28.5)	**<0.001**	18.42 (11.36 - 30)	**<0.001**	27.62 (14.72 - 51.83)	**<0.001**	26.82 (13.98 - 51.5)	**<0.001**
	IA1	IIIA	44.08 (27.29 - 71.2)	**<0.001**	31.6 (19.1 - 52.29)	**<0.001**	69.83 (36.62 - 133.2)	**<0.001**	47.69 (24.44 - 93.1)	**<0.001**
	IA1	IIIB	43.59 (27.57 - 68.9)	**<0.001**	34.48 (21.3 - 55.9)	**<0.001**	69.5 (37.13 - 130.1)	**<0.001**	51.91 (27.07 - 99.6)	**<0.001**
	IA1	IVA	64.41 (40.28 - 103)	**<0.001**	37.4 (22.83 - 61.3)	**<0.001**	101.13 (53.48 - 191)	**<0.001**	55.23 (28.54 - 107)	**<0.001**
	IA1	INOS	11.23 (6.89 - 18.3)	**<0.001**	9.96 (6.07 - 16.36)	**<0.001**	13.67 (7.04 - 26.55)	**<0.001**	11.51 (5.88 - 22.52)	**<0.001**
	IA1	IINOS	25.41 (10.15 - 63.6)	**<0.001**	20.74 (8.17 – 52.6)	**<0.001**	39.84 (13.62 - 117)	**<0.001**	33.17 (11.17 - 98.5)	**<0.001**
	IA1	IIINOS	45.83 (27.29 - 77)	**<0.001**	31.38 (18.28 - 54)	**<0.001**	73.62 (37.33 - 145)	**<0.001**	46.93 (23.28 - 94.6)	**<0.001**
LVSI	No	Yes	3.19 (2.92 - 3.49)	**<0.001**	1.43 (1.28 - 1.6)	**<0.001**	3.68 (3.33 - 4.07)	**<0.001**	1.55 (1.37 - 1.76)	**<0.001**
	No	UNK	4.36 (3.51 - 5.41)	**<0.001**	1.4 (1.12 - 1.76)	**0.0037**	5.18 (4.11 - 6.53)	**<0.001**	1.62 (1.26 - 2.07)	**<0.001**
Primary site surgery	No	Yes	0.28 (0.26 - 0.32)	**<0.001**	NA (NA - NA)	NA	0.29 (0.26 - 0.32)	**<0.001**	NA	NA
RLNS	No	Yes	0.27 (0.24 - 0.3)	**<0.001**	0.6 (0.44 - 0.82)	**0.0013**	0.28 (0.25 - 0.31)	**<0.001**	0.73 (0.52 - 1.03)	0.0768
	No	UNK	0.78 (0.5 - 1.21)	0.263	1.19 (0.72 - 1.97)	0.5	0.89 (0.56 - 1.42)	0.619	1.39 (0.81 - 2.4)	0.2335
Radiation	Beam radiation	brachytherapy	0.73 (0.23 - 2.25)	0.579	0.9 (0.29 - 2.8)	0.8495	0.59 (0.15 - 2.37)	0.458	0.78 (0.19 - 3.15)	0.7293
	Beam radiation	BRB	0.65 (0.59 - 0.72)	**<0.001**	0.68 (0.61 - 0.76)	**<0.001**	0.65 (0.58 - 0.72)	**<0.001**	0.7 (0.62 - 0.79)	**<0.001**
	Beam radiation	No/UNK	0.32 (0.28 - 0.35)	**<0.001**	1.09 (0.94 - 1.26)	0.24	0.29 (0.26 - 0.33)	**<0.001**	1.04 (0.88 - 1.22)	0.6586

HR, hazard Ratio; CI, conﬁdence interval; P<0.05 is marked with bold black. NA, not applicable.

### Construction of prognostic nomograms

**Figure 1:**
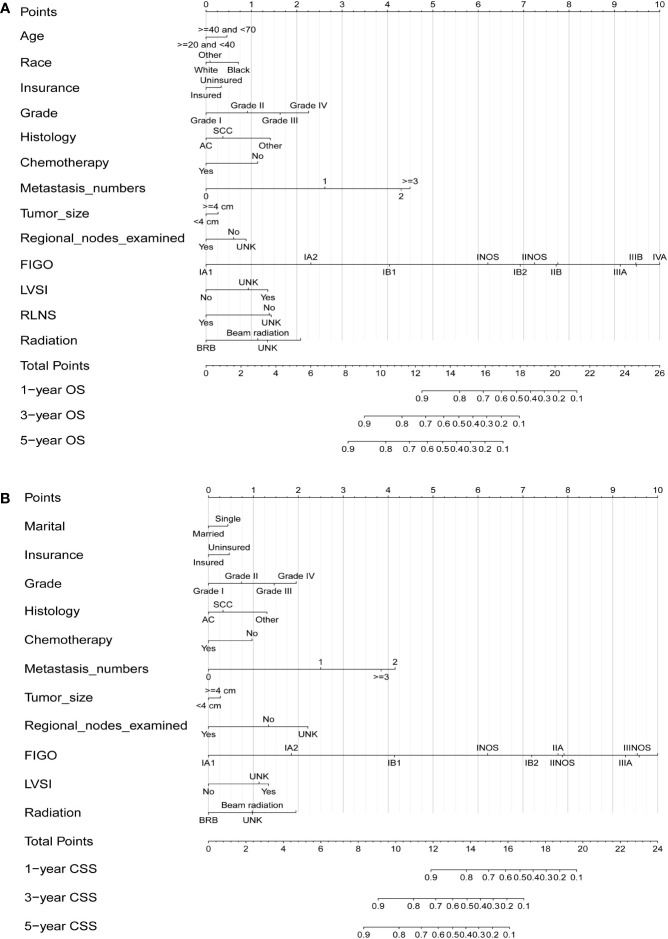
Nomograms for predicting 1-, 3-, and 5-year OS **(A)** and CSS **(B)** in patients with cervical cancer.

After selecting the minimum AIC value, the above-mentioned parameters were used to develop nomograms for predicting 1-, 3-, and 5-year OS and CSS (**Figure 1**). Each variable was given a score based on the corresponding point on the “point axis”. Next, we added the scores of all variables to obtain a total score, and then drew a vertical line from the “total point axis” to the corresponding “survival axis” to estimate the predicted probability of 1-, 3-, and 5-year OS and CSS. According to the nomograms, we concluded that FIGO stage made the largest contribution to the predicted probability, followed by metastasis and grade respectively.

### Validation of the nomograms

**Figure 2:**
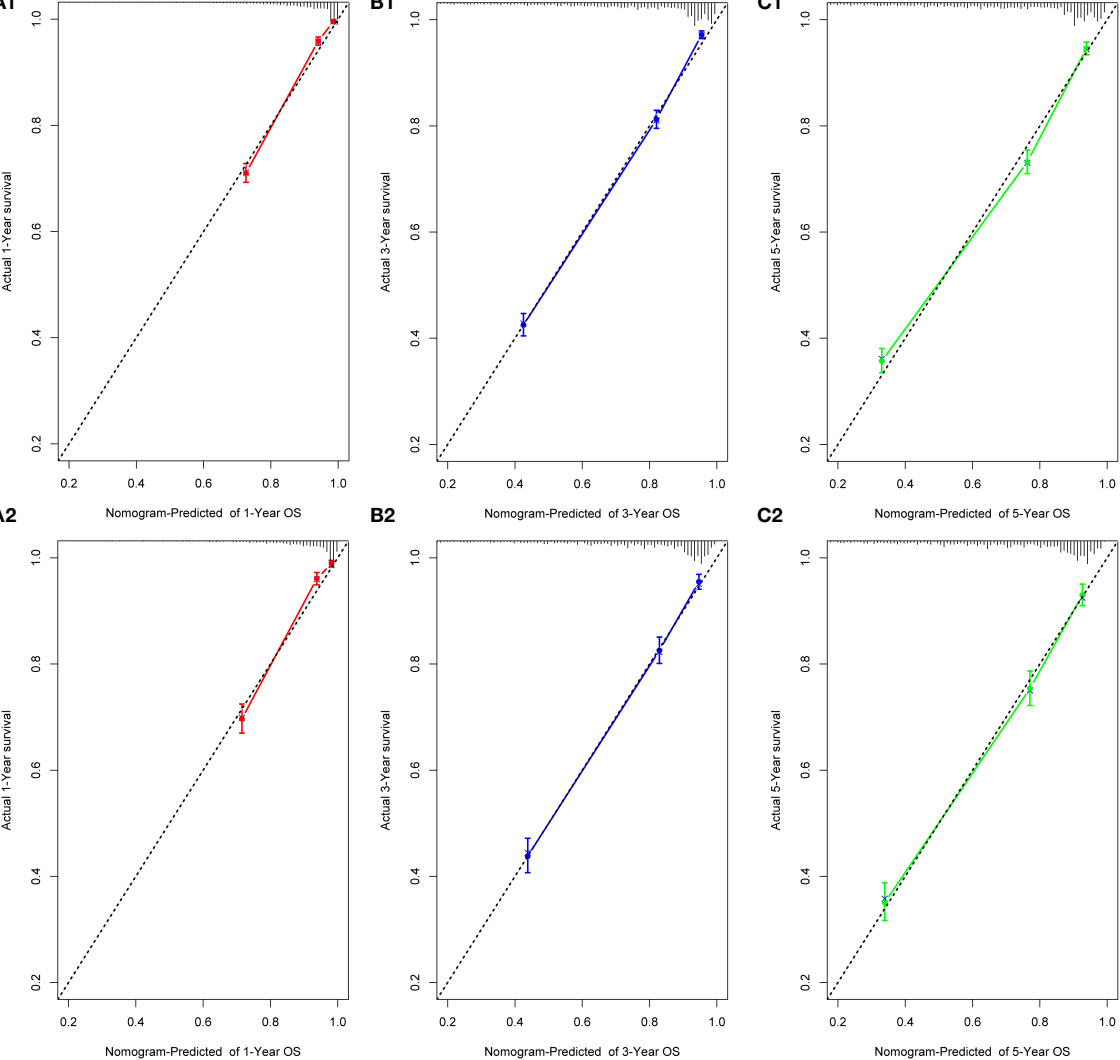
Calibration plots for 1-, 3-, and 5-year OS prediction for the training cohort (**A1, B1, C1)** and validation cohort **(A2, B2, C2)**.

**Figure 3:**
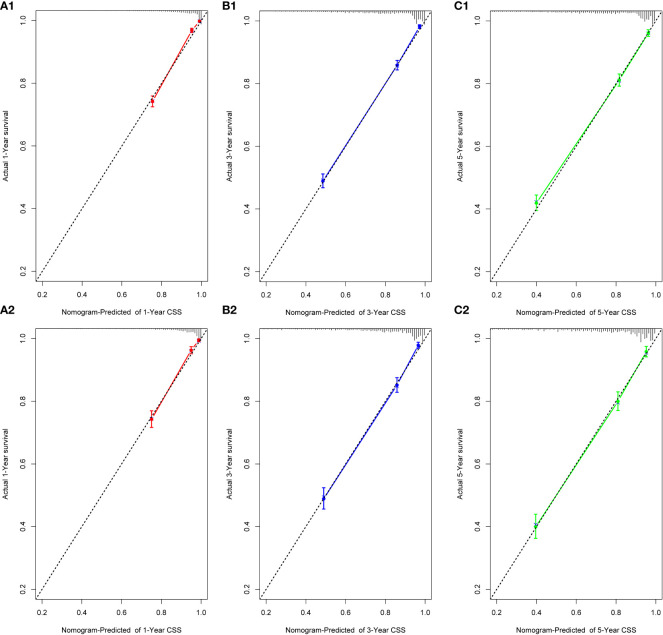
Calibration plots for 1-, 3-, and 5-year CSS prediction for the training cohort **(A1, B1, C1)** and validation cohort **(A2, B2, C2)**.

We performed internal training and external validation on the nomograms using different cohorts. In the internal cohort, we obtained C-index values of 0.831 (95% CI, 0.823–0.839) for prediction of OS, and 0.844 (95% CI, 0.836–0.852) for prediction of CSS. In the external validation cohort, we obtained C-index values of 0.824 (95% CI, 0.810–0.838) for OS and 0.841 (95% CI, 0.827–0.855) for CSS. The calibration plots for the nomograms showed that the predictions of OS (**Figure 2**) and CSS (**Figure 3**) made by the 1-, 3-, and 5-year survival probability models were almost consistent with actual observations, in both the internal and external cohorts.

**Figure 4:**
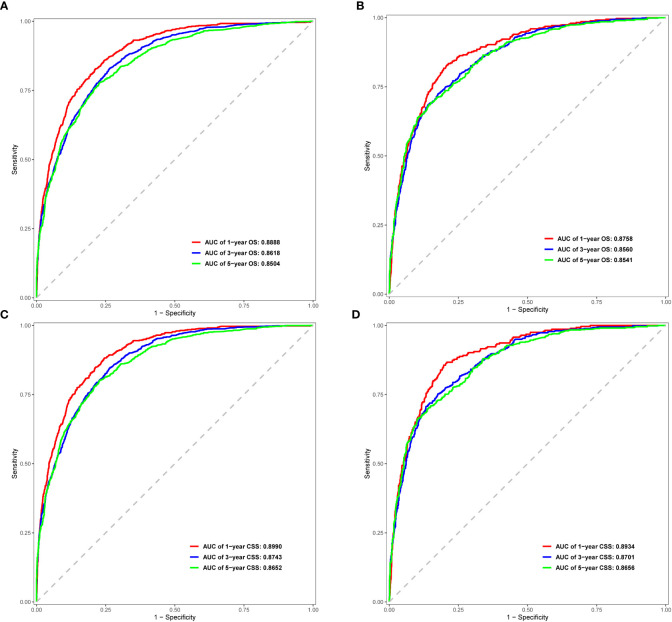
ROC curves for 1-, 3-, and 5-year OS and CSS in the training cohort **(A, C)** and validation cohort **(B, D)**.

In the ROC curve analysis of the models, the area under the curve (AUC) values for prediction of 1-, 3-, and 5-year OS were 0.8888, 0.8618, and 0.8504 in the internal cohort, and 0.8758, 0.8560, and 0.8541 in the external cohort, respectively (**Figures 4A, C**). For prediction of 1-, 3-, and 5-year CSS, the AUC values were 0.8990, 0.8743, and 0.8652 in the training cohort, and 0.8934, 0.8701, and 0.8656 in the validation cohort, respectively (**Figures 4B, D**). The validation of these two nomograms demonstrated the excellent predictive accuracy for OS and CSS based on C-index and AUC.

### Clinical applicability

**Figure 5:**
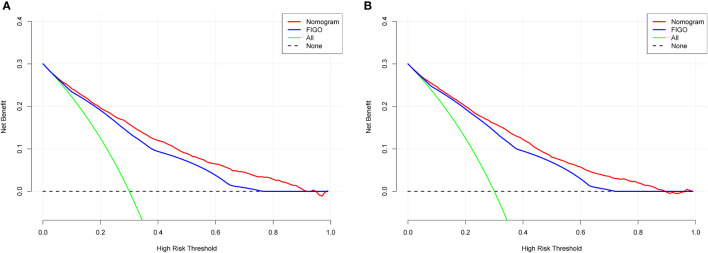
Decision curve analysis of the nomogram and FIGO stage prediction model for predicting OS **(A)** and CSS **(B)** in the training cohort. The x-axis represents the percentage of threshold probability, whereas the y-axis represents the net benefit, calculated by adding the true positives and subtracting the false positives.

**Figure 6:**
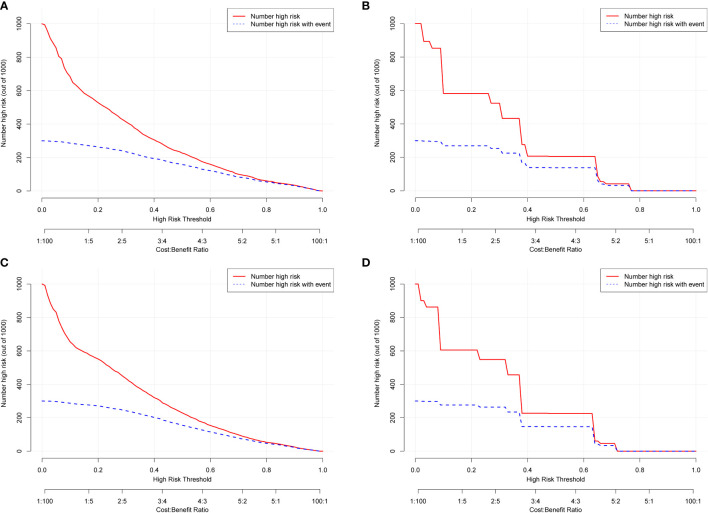
Clinical impact curves (CICs) for OS and CSS of the nomogram **(A, C)** and FIGO stage **(B, D).** The red curve (number of high-risk individuals) indicates the number of people who were classified as positive (high risk) by the model at each threshold probability; the blue curve (number of high-risk individuals with outcome) is the number of true positives at each threshold probability. The CICs provided visual confirmation of the high net clinical benefit of the nomograms and confirmed the clinical value of the model.

DCA was used to evaluate the clinical applicability of the nomograms (17). **Figure 5** shows decision curves for the nomograms and the FIGO stage for OS and CSS. These indicated that our model was superior to the FIGO stage, providing greater net clinical benefit with a threshold probability between 0 and 90%. CIC analysis (**Figure 6**) was performed to evaluate the clinical applicability of the risk prediction nomograms (18, 19) and FIGO stage. The DCA and CICs showed that the nomograms had greater net benefit within wide and practical ranges of threshold probabilities and impacted patient outcomes, indicating that our models have a significant predictive value.

### Survival outcomes

During the follow-up period, the rates of OS- and CSS-related adverse events were 26% (2882/11148) and 21% (2332/11148), respectively. Analysis of survival outcomes (**Table 3**) showed that the 1-, 3-, and 5-year OS rates in the training cohort were 88.8, 73.5, and 67.7%, whereas those in the validation cohort were 88.1%, 73.9%, and 67.7%, respectively. The 1-, 3-, and 5-year CSS rates in the training cohort were 90.4%, 78.0%, and 73.6%, and those in the validation cohort were 90.1%, 77.7%, and 72.4%, respectively.

**Table 3:** Survival analysis of OS and CSS in the training cohort and validation cohort.

Endpoints	Months	n.risk	n.event	Survival	std.err	95% CI
OS of training cohort	12	6619	851	0.888	0.0036	0.881 - 0.895
	36	3375	937	0.735	0.0055	0.725 - 0.746
	60	1453	197	0.677	0.0065	0.665 - 0.690
OS of validation cohort	12	2819	384	0.881	0.0057	0.870 - 0.893
	36	1445	375	0.739	0.0084	0.722 - 0.755
	60	629	89	0.677	0.0100	0.657 - 0.696
CSS of training cohort	12	6619	724	0.904	0.0034	0.897 - 0.910
	36	3375	735	0.780	0.0052	0.770 - 0.791
	60	1453	142	0.736	0.0062	0.724 - 0.748
CSS of validation cohort	12	2819	317	0.901	0.0053	0.891 - 0.911
	36	1445	314	0.777	0.0080	0.761 - 0.793
	60	629	72	0.724	0.0097	0.705 - 0.743

**Figure 7:**
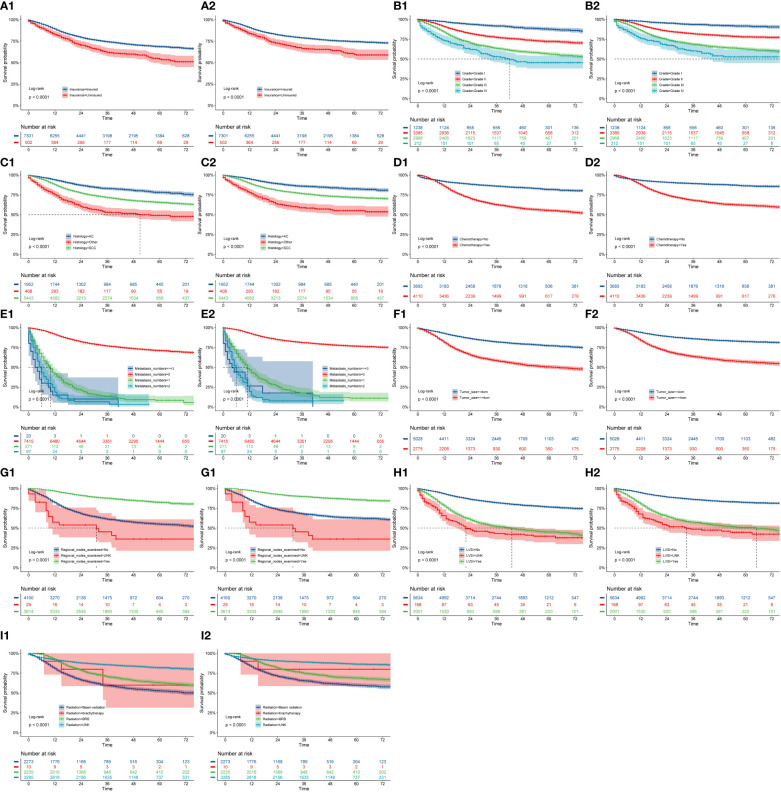
OS and CSS stratified by patient characteristics in the training cohort: **(A1, A2)** insurance; **(B1, B2)** grade; **(C1, C2)** history; **(D1, D1)** chemotherapy; **(E1, E2)** metastasis numbers; **(F1, F2)** tumor size; **(G1, G2)** regional nodes examined; **(H1, H2)** LVSI; **(I1, I2)** radiation.

We used the nine prognostic factors to visually present OS and CSS in the training cohort (**Figure 7****)**. The CC patients with insurance had better survival outcomes (**Figures 7A1, A2**). OS and CSS decreased significantly with increasing grade (**Figures 7B1, B2**); that is, the higher the pathological grade, the worse the degree of differentiation and the higher the degree of malignancy. Patients with SCC had worse OS and CSS compared with patients with AC(adenocarcinoma) histopathology (**Figures 7C1, C2**). Patients who did not undergo chemotherapy treatment had obviously better survival outcomes in terms of both OS and CSS than those that received chemotherapy (**Figures 7D1, D2**). Patients diagnosed with metastasis (**Figures 7E1, E2**) or tumor size greater than 4 cm (**Figures 7F1, F2**) had worse survival. Regional lymph nodes with examination (**Figures 7G1, G2**) and those without positive lymph nodes (**Figures 7H1, H2**) were associated with better prognosis. Compared with beam radiation, BRB treatment had better survival outcomings (**Figures 7I1, I2**).

## Discussion

Among female malignant tumors, CC ranks fifth in incidence and seventh in mortality worldwide (2022 Cancer Report). Owing to improvements in health awareness, early diagnosis, and early treatment, the incidence and mortality of CC have improved in developed countries; however, the early clinical symptoms of cervical cancer are not obvious, and the disease is usually locally advanced at first diagnosis. Comprehensive treatment of CC in the early stage is mainly based on surgery, and radiotherapy has a pivotal role in the treatment of patients with locally advanced stage disease. Global large-scale prospective randomized controlled clinical trials of concurrent chemoradiotherapy and radiotherapy alone in the treatment of CC have clarified the normative status of concurrent chemoradiotherapy (20–22).

Although technologies for CC treatment have become increasingly advanced, and the use of surgery and concurrent chemoradiotherapy have enabled curative effects in more patients, 20–40% of patients with CC still experience recurrence or metastasis within 2 years (23), with a recurrence rate within 3 years after radiotherapy that exceeds 70% (24). Therefore, there is an urgent need for a more accurate and effective method to evaluate OS and CSS in patients with CC. According to studies of CC at different FIGO stages, failure rates of local treatment in patients with stage IB, IIA, IIB, III, and IV CC were 10%, 17%, 23%, 42%, and 74%, respectively.

In recent years, increasing numbers of studies have focused on the use of predictive models to improve survival, although bottlenecks and deficiencies exist. As a novel, simple, and direct prediction model, the nomogram can directly visualize predicted OS and CSS and provide a reference for further examination and clinical decision-making. In our study, factors including age, race, marital status, insurance status, grade, histology, chemotherapy, metastasis number, tumor size, regional node examination, FIGO stage, LVSI, RLNS, and radiation showed associations with prognosis in patients with CC, and we built nomograms for both OS and CSS based on these factors. Finally, nomograms were developed to calculate the probabilities of 1-, 3-, and 5-year OS (based on 13 independent prognostic factors) and CSS (based on 11 independent prognostic factors) in patients with CC. Our nomograms indicated that FIGO stage made the largest contribution to the predicted probability between 1-, 3-, and 5-year OS and CSS, which was consistent with a large body of previous research (6, 9, 25).

We analyzed the survival outcomes (OS and CSS) of patients stratified by the following factors: insurance status, grade, histology, chemotherapy, metastasis number, tumor size, regional node examination, LVSI, and radiation. Patients with insurance had better survival outcomes, and this has not been reported by previous studies (6–9). CC patients with AC histopathology had slightly better prognoses than those with SCC; similarly, this result has rarely been reported in previous studies (8, 9, 26, 27). Only about 45% of the patients with CC benefited from initial regional lymph node examination. It had been reported that LVSI was an important poor prognostic factor for patients with early cervical cancer. Diffuse lymphatic involvement (diffuse LVSI) has predictive significance for the survival prognosis of patients compared with focal or non-focal lesions (28). In our study, patients with LVSI had worse OS and CSS (P<0.001). In clinical practice, radiation and chemotherapy are the most commonly used effective treatments for patients with CC and lead to significant improvements in survival time (7). Our results indicated that CC patients without chemotherapy treatment had better prognoses than those that received chemotherapy. Compared with beam radiation, patients who chose BRB had a more favorable survival outcome (13).

Surgery is still the main treatment method for early cervical cancer, particularly laparoscopic minimally invasive surgery is currently a popular surgical method. However, combined with the prospective large-scale clinical study LACC trial (Laparoscopic Approach to Cervical Cancer) and meta-analysis (29, 30), it was concluded that this surgical method did not benefit the survival of these patients. In our study, we mainly focus on RSS, primary site surgery and RLNS. In the univariate Cox regression, the three variables were significant for both OS and CSS (P<0.001), in the multivariate Cox regression, they had no significant for both OS and CSS (P>0.05). Since there is no detailed record of specific surgical approaches, more prospective studies may be needed to further determine the safety and efficacy of minimally invasive surgery.

Survival and prognosis studies on patients with locally advanced cervical cancer showed that (31), multivariate analysis of tumors ≥6 cm had worse loco-regional-recurrence-free survival (LRFS) and OS; AC and positive lymph nodes were associated with distant-metastasis-free survival (DMFS); adjuvant chemotherapy has longer DMFS and OS. In our study, tumor size ≥4 cm were poor prognostic factors for OS and CSS; patients with AC had better OS and CSS; those with positive lymph nodes and a history of chemotherapy had worse OS and CSS, because the data source did not clearly classify neoadjuvant chemotherapy or adjuvant chemotherapy, so more follow-up studies are needed to confirm the contribution of chemotherapy timing.

Previously, many studies developed nomograms for diagnosis and prognostic prediction in patients with CC; however, these studies had many limitations, including insufficient sample size (7), the model having a low C-index and prediction accuracy (6, 32), insufficient inclusion and exclusion criteria (6, 33), and a single study endpoint (8, 9). To our knowledge, compared with other studies evaluating CC survival using nomograms (6–9), the present study considered more real-time sample data and a more complete set of patient prognostic factors (13) than previous studies, showed excellent predictive accuracy for OS and CSS (6–9, 33), and demonstrated greater clinical net benefit. The calibration plots were almost consistent with actual observations, and we obtained excellent C-index (approximately 0.84) and AUC (approaching 0.89) values, indicating that our nomograms showed outstanding performance in predicting 1-, 3-, and 5-year OS and CSS. In addition, we compared FIGO staging and the nomograms with respect to their net benefit. FIGO stage is widely used clinically, and the use of a nomogram could reduce the diversity due to different treatments and sociodemographic statuses when predicting the prognosis of patients with CC (25, 32). We found that the nomograms had greater net benefit than the FIGO stage according to DCA and CIC. Therefore, our nomograms could represent a reliable alternative or supplementary tool to predict survival outcomes.

Our study included the latest and sufficient data sets through the SEER database, which contained enough clinical and non-clinical factors to have better practical significance in line with the real world. We constructed more 10 independent prognostic factors nomograms to calculate the probabilities of 1-, 3-, and 5-year OS (13 factors) and CSS (11 factors) in patients with CC. To provide strong evidence, we evaluated our model internally and externally using five approaches: the C-index, ROC, calibration plots, DCA, and CICs, which made our study more complete and reliable compared with previous studies. From the 1-, 3-, and 5-year OS and CSS nomograms, it was found that the scores of FIGO stage, metastasis number, and grade were the three highest in all indicators, which can provide direct and effective actual clinical implications for survival prognostic assessment. However, this study had some limitations. First, this was a retrospective population analysis without multicenter validation data. Second, there was a lack of information about other important factors, including HPV infection status and blood type parameters; future studies could refine the nomograms by incorporating these predictors. Third, our data were from the US population only, and the demographic data was relatively homogeneous. Future analyses of multicenter data with larger sample sizes, more variables including clinical and non-clinical factors, and patients of different ethnicities are required to validate our conclusions.

## Conclusions

We used the SEER database to analyze prognostic data for CC patients, identified independent prognostic factors, and constructed nomograms for estimating 1-, 3-, and 5-year OS and CSS. Internal and external validation showed that these models had excellent predictive performance. They could thus be considered as reliable tools to predict prognosis, which is essential for maximizing the patient’s chance of survival.

## Data availability statement

Publicly available datasets were analyzed in this study. The data can be found at SEER National Cancer Institute (via accession number: 19279-Nov2020).

## Author contributions

KJ , YL and YA conceived and designed the study. The YL and LJ provided administrative and funding support. KJ and YL collected and assembled the data. KJ and YL contributed to data processing, interpretation of results, and drafting of the manuscript. All authors read and approved the manuscript.

## Funding

This study was supported by the Scientific Research Fund project of Yunnan Education Department (2022J0265). This research was funded by the National Natural Science Foundation of China under Grant No. 62262035.

## Acknowledgments

We thank the Third Affiliated Hospital of Kunming Medical University for supporting the writing of this manuscript.

## Conflict of interest

The authors declare that the research was conducted in the absence of any commercial or financial relationships that could be construed as a potential conflict of interest.

## Publisher’s note

All claims expressed in this article are solely those of the authors and do not necessarily represent those of their affiliated organizations, or those of the publisher, the editors and the reviewers. Any product that may be evaluated in this article, or claim that may be made by its manufacturer, is not guaranteed or endorsed by the publisher.

## References

[1] Siegel RL Miller KD Fuchs HE Jemal A. Cancer statistics, 2021. CA Cancer J Clin (2021) 71(1):7–33. doi: 10.3322/caac.21654 33433946

[2] Islami F Fedewa SA Jemal A. Trends in cervical cancer incidence rates by age, race/ethnicity, histological subtype, and stage at diagnosis in the united states. Prev Med (2019) 123:316–23. doi: 10.1016/j.ypmed.2019.04.010 31002830

[3] Saslow D Andrews KS Manassaram–Baptiste D Smith RA Fontham ETH. Human papillomavirus vaccination 2020 guideline update: American cancer society guideline adaptation. CA Cancer J Clin (2020) 70(4):274–80. doi: 10.3322/caac.21616 32639044

[4] Fontham ETH Wolf AMD Church TR Etzioni R Flowers CR Herzig A. Cervical cancer screening for individuals at average risk: 2020 guideline update from the American cancer society. CA Cancer J Clin (2020) 70(5):321–46. doi: 10.3322/caac.21628 32729638

[5] Waggoner SE. Cervical cancer. Lancet (2003) 361(9376):2217–25. doi: 10.1016/s0140-6736(03)13778-6 12842378

[6] Li Z Lin Y Cheng B Zhang Q Cai Y. Prognostic model for predicting overall and cancer–specific survival among patients with cervical squamous cell carcinoma: a SEER based study. Front Oncol (2021) 11:651975. doi: 10.3389/fonc.2021.651975 34336651 PMC8317021

[7] Zhang S Wang X Li Z Wang W Wang L. Score for the overall survival probability of patients with first–diagnosed distantly metastatic cervical cancer: a novel nomogram–based risk assessment system. Front Oncol (2019) 9:1106. doi: 10.3389/fonc.2019.01106 31750238 PMC6848257

[8] Wang C Yang C Wang W Xia B Li K Fet S. A prognostic nomogram for cervical cancer after surgery from SEER database. J Cancer (2018) 9(21):3923–8. doi: 10.7150/jca.26220 PMC6218784 30410596

[9] Xie G Wang R Shang L Qi C Yang L Huang L. Calculating the overall survival probability in patients with cervical cancer: a nomogram and decision curve analysis–based study. BMC Cancer (2020) 20(1):833. doi: 10.1186/s12885-020-07349-4 32873257 PMC7466454

[10] Balachandran VP Gonen M Smith JJ DeMatteo RP. Nomograms in oncology: more than meets the eye. Lancet Oncol (2015) 16:173–80. doi: 10.1016/S1470-2045(14)71116-7 PMC4465353 25846097

[11] Song Z Wang Y Zhou Y Zhang D . A Novel Predictive Tool for Determining the Risk of Early Death From Stage IV Endometrial Carcinoma: A Large Cohort Study. Front Oncol (2020) 10:620240. doi: 10.3389/fonc.2020.620240 33381462 PMC7769006

[12] Iasonos A Schrag D Raj GV Panageas KS. How to build and interpret a nomogram for cancer prognosis. J Clin Oncol (2008) 26(8):1364–70. doi: 10.1200/jco.2007.12.9791 18323559

[13] Tian T Gong X Gao X Li Y Ju W Ai Y. Comparison of survival outcomes of locally advanced cervical cancer by histopathological types in the surveillance, epidemiology, and end results (SEER) database: a propensity score matching study. Infect Agent Cancer (2020) 15:33. doi: 10.1186/s13027-020-00299-3 32435273 PMC7222537

[14] Liang W Yang P Huang R Xu L Wang J Liu W. A combined nomogram model to preoperatively predict histologic grade in pancreatic neuroendocrine tumors. Clin Cancer Res (2019) 25(2):584–94. doi: 10.1158/1078-0432.Ccr-18-1305 30397175

[15] Vickers AJ Elkin EB. Decision curve analysis: a novel method for evaluating prediction models. Med Decis Making (2006) 26:565–74. doi: 10.1177/0272989X06295361 PMC2577036 17099194

[16] Tolles J Meurer WJ. Logistic regression: relating patient characteristics to outcome. JAMA (2016) 316(5):533–4. doi: 10.1001/jama.2016.7653 27483067

[17] Van Calster B Wynants L Verbeek JFM Verbakel JY Christodoulou E Vickers AJ. Reporting and interpreting decision curve analysis: a guide for investigators. Eur Urol (2018) 74(6):796–804. doi: 10.1016/j.eururo.2018.08.038 30241973 PMC6261531

[18] Qi Y Wu S Tao L Xu G Chen J Feng Z. A population–based study: how to identify high–risk T1–2 esophageal cancer patients? Front Oncol (2021) 11:766181. doi: 10.3389/fonc.2021.766181 34966675 PMC8710781

[19] Zhu YJ Chen Y Hu HY Zhou YW Zhu YT Liu JY. Predictive risk factors and online nomograms for synchronous colon cancer with liver metastasis. Front Oncol (2020) 10:. doi: 10.3389/fonc PMC7566411 33123459

[20] Morris M Eifel PJ Lu J Grigsby PW Levenback C Stevens RE. Pelvic radiation with concurrent chemotherapy compared with pelvic and para–aortic radiation for high–risk cervical cancer. N Engl J Med (1999) 340(15):1137–43. doi: 10.1056/NEJM199904153401501 10202164

[21] Peters WA Liu PY Barrett RJ Stock RJ Monk BJ Berek JS. Concurrent chemotherapy and pelvic radiation therapy compared with pelvic radiation therapy alone as adjuvant therapy after radical surgery in high–risk early–stage cancer of the cervix. J Clin Oncol (2000) 18(8):1606–13. doi: 10.1200/JCO.2000.18.8.1606 10764420

[22] Thomas GM. Improved treatment for cervical cancer–concurrent chemotherapy and radiotherapy. N Engl J Med (1999) 340(15):1198–200. doi: 10.1056/NEJM199904153401509 10202172

[23] Peiretti M Zapardiel I Zanagnolo V Landoni F Morrow CP Maggioni A. Management of recurrent cervical cancer: a review of the literature. Surg Oncol (2012) 21(2):e59–66. doi: 10.1016/j.suronc.2011.12.008 22244884

[24] Goncalves A Fabbro M Lhommé C Gladieff L Extra JM Floquet A. A phase II trial to evaluate gefitinib as second– or third–line treatment in patients with recurring locoregionally advanced or metastatic cervical cancer. Gynecol Oncol (2008) 108(1):42–6. doi: 10.1016/j.ygyno.2007.07.057 17980406

[25] Tang X Guo C Liu S Guo J Hua K Qiu J. A novel prognostic nomogram utilizing the 2018 FIGO staging system for cervical cancer: a large multicenter study. Int J Gynaecol Obstet (2021) 155(1):86–94. doi: 10.1002/ijgo.13644 33587753

[26] Yang J Tian G Pan Z Zhao F Feng X Liu Q. Nomograms for predicting the survival rate for cervical cancer patients who undergo radiation therapy: a SEER analysis. Future Oncol (2019) 15(26):3033–45. doi: 10.2217/fon-2019-0029 31452393

[27] Liu Q Li W Xie M Yang M Xu M Yang L. Development and validation of a SEER–based prognostic nomogram for cervical cancer patients below the age of 45 years. Bosn J Basic Med Sci (2021) 21(5):620–31. doi: 10.17305/bjbms.2020.5271 PMC8381204 33485294

[28] Ronsini C Anchora LP Restaino S Fedele C Arciuolo D Teodorico E. The role of semiquantitative evaluation of lympho–vascular space invasion in early stage cervical cancer patients. Gynecol Oncol (2021) 162(2):299–307. doi: 10.1016/j.ygyno.2021.06.002 34116834

[29] Ramirez PT Frumovitz M Pareja R Lopez A Vieira M Ribeiro R. Minimally invasive versus abdominal radical hysterectomy for cervical cancer. N Engl J Med (2018) 379(20):1895–904. doi: 10.1056/NEJMoa1806395 30380365

[30] Ronsini C Köhler C De Franciscis P La Verde M Mosca L Solazzo MC. Laparo–assisted vaginal radical hysterectomy as a safe option for minimal invasive surgery in early stage cervical cancer: A systematic review and meta–analysis. Gynecol Oncol (2022) 166(1):188–95. doi: 10.1016/j.ygyno.2022.04.010 35513934

[31] Queiroz ACM Fabri V Mantoan H Sanches SM Guimarães APG Ribeiro ARG. Risk factors for pelvic and distant recurrence in locally advanced cervical cancer. Eur J Obstet Gynecol Reprod Biol (2019) 235:6–12. doi: 10.1016/j.ejogrb.2019.01.028 30771718

[32] Zang L Chen Q Zhang X Zhang X Chen J Fang Y. Nomogram predicting overall survival in patients with FIGO II to III squamous cell cervical carcinoma under radical radiotherapy: A retrospective analysis based on 2018 FIGO staging. Cancer Manag Res (2021) 13:9391–400. doi: 10.2147/CMAR.S336892 PMC8722567 35002316

[33] Jiang AG Cai X. Construction and validation of the prognostic model for patients with neuroendocrine cervical carcinoma: a competing risk nomogram analysis. BMC Cancer (2022) 22(1):4. doi: 10.1186/s12885-021-09104-9 34980030 PMC8722105

